# The past, present, and future of Alzheimer's disease—part 3: the future

**DOI:** 10.1055/s-0046-1824435

**Published:** 2026-07-14

**Authors:** Ricardo Nitrini, Leonel Tadao Takada, Sonia Maria Dozzi Brucki

**Affiliations:** 1 Universidade de São Paulo, Faculdade de Medicina, Departamento de Neurologia, Grupo de Neurologia Cognitiva e Comportamental, São Paulo SP, Brazil.

**Keywords:** Alzheimer Disease, Biomarkers, Therapeutics, Genetics, Inflammation, Geroscience

## Abstract

Predicting future advances in science is practically impossible because unexpected discoveries can radically change the march of ongoing processes. Predicting the nearest future may have a slightly greater chance of success. First, the diagnosis of risk factors for Alzheimer's disease (AD), based on the preponderant role of genetics, will be made very early. Diagnosis will be made with biomarkers prior to clinical manifestations. Furthermore, the prevention of AD and of dementia due to AD will be possible with better control of the many risk factors for dementia, healthier lifestyles, multi-target drugs, and genetic therapy. The relationship between AD and aging will be better understood, opening possibilities for intervention in aging itself. As the pathophysiology of AD is not unique, treatment will be individualized, according to the stage and characteristics of the disease. Most clinical trials will have surrogate markers as primary outcomes. Treatments to decrease the velocity or arrest the evolution of mild cognitive impairment (MCI) and mild dementia caused by AD will be available. Additionally, artificial intelligence (AI) will aid in the diagnosis and selection of the best treatments. Access to the new methods for diagnosing and treating AD is currently limited to a small parcel of the population, especially in low- and middle-income countries, because of their high costs. Due to the high prevalence of AD, it is possible these will become less expensive in the future. The possibility of reversing dementia once it has set in will probably have to wait for longer.

## INTRODUCTION

Predicting the future in a particular field of science is practically impossible, as unexpected discoveries can radically change ongoing processes and perspectives. Neurologists who predicted the future of neurology in the middle of the last century were too conservative because they could not imagine the advances and the impact of neuroimaging, information technology (IT), and the internet. Now, we are facing artificial intelligence (AI) and an even more intangible future is ahead of us. One may have a slightly greater chance of success at predicting the nearest future.

Most neuroscientists can write articles on this same topic with more information about forthcoming paths. As clinicians, we will focus on future challenges regarding diagnosis and new treatments.

## NEAREST FUTURE OF ALZHEIMER'S DISEASE (AD)

This is a very exciting moment in the field of cognitive and behavioral neurology, particularly for Alzheimer's disease (AD) diagnosis and treatment. This is a period in which novel AD biomarkers are being discovered or improved, as well as more widely used in clinical practice. Furthermore, novel disease-modifying treatments are being released in the market (though not without some controversy). 1 2 Let us focus on these recent discoveries to glimpse the near future.

## DIAGNOSIS

Recently, there has been a major change in the diagnostic criteria for AD. 3 According to these criteria, dementia is no longer necessary for its diagnosis, which is considered a continuum with a long asymptomatic phase when diagnosis is only possible with biomarkers. 3 There is no doubt that this is the natural path for the future of AD. However, the difference between the asymptomatic phase of AD and the dementia due to AD is too great for doctors to keep using the same diagnosis in current clinical practice, without this new concept of AD having been widely disseminated to the entire community. 4 5 This difference and these new concepts will be widely disseminated to the population in the coming years. 5 We will address this issue in more detail when discussing the timing of diagnosis.

### Clinical diagnosis

Diagnosis of the stages of the AD continuum depends and will continue to depend on clinical assessment, with the addition of biomarkers. More effective treatments will define the importance of diagnosis in all its stages: asymptomatic cases, subjective cognitive decline (SCD), mild cognitive impairment (MCI), as well as mild, moderate, or severe dementia due to AD.

Computerized unsupervised remote test batteries aiming to reach a larger number of individuals with early cognitive changes have been designed, 6 7 and will be important in near future. Patient-centered testing platforms, which allow individuals to complete cognitive assessments when it is convenient for them in their own devices (cellphone or PC) will be available. 6 7

### Neuroimaging

Magnetic resonance imaging (MRI) and positron emission tomography with fluor-deoxyglucose positron-emission tomography (FDG-PET) have been very important for identifying changes to the brain structure and metabolism, for confirming the diagnosis with amyloid PET, and for evaluating disease stages, particularly with tau-PET. 8

Neuroimaging biomarkers for synaptic loss are under research and will be available for clinical diagnosis, 9 and as surrogate markers. Synaptic loss due to reduced neuroplasticity 10 is the major correlate of cognitive impairment in AD. 11

Inflammatory changes in AD have been detected by neuroimaging biomarkers, 12 and are presented further in the text.

New tracers will be available for diagnosing other tauopathies, identifying deposits of alpha-synuclein, TDP-43, blood-brain barrier (BBB) disruptions, and glymphatic brain disorders, among others. As AD is frequently associated with other degenerative or vascular diseases, mainly in individuals over the age of 80 years, 13 new tracers are needed.

But the high cost may make the use of neuroimaging unfeasible in clinical practice, particularly for low-middle income countries. Changes to reduce costs are already underway, such as the production of less expensive MRI and PET scanners dedicated only to the brain. 14 15

### Plasma biomarkers and other biomarkers

Plasma biomarkers of the presence in the brain of Aβ protein and phospho-tau protein (ptau) will be the most widely used tools for early diagnosis of AD. A tau protein biomarker in plasma (eMTBR-tau243) has a good capacity for biological staging of this disease. 16

Other plasma biomarkers that may act as surrogate markers of evolution will be discovered in the near future. Plasma biomarkers of synaptic degeneration such as neurogranin, BACE1, synaptotagmin, SNAP-25, GAP-43, synaptophysin, and others will be available. 17

TDP-43 and alpha-synuclein are frequently found proteins in the brains of patients with Alzheimer's disease, particularly in older individuals. These proteins contribute to cognitive decline and dementia, yet there are currently no biomarkers for them. Such biomarkers will become available in near future.

#### Cognitive networks

TDP-43 and alpha-synuclein are frequently found proteins in the brains of patients with Alzheimer's disease, particularly in older individuals. These proteins contribute to cognitive decline and dementia, yet there are currently no biomarkers for them. Such biomarkers will become available in near future. In AD, there is progressive involvement of neural networks. Currently, the involvement of these networks is recognized through clinical and neuropsychological evaluations, and FDG-PET. Functional MRIs are mostly used in research, but other more friendly methods to detect impaired synchrony in cortical networks will be available.

### Genetics in the diagnosis of AD

In the future, it will be possible to use genetics to predict the risk of AD. Very early risk diagnosis probably will be possible by calculating the polygenic risk score (PRS). Let us briefly go over the genetics of AD.

Since 1980, genetic studies have allowed the development of the amyloid cascade hypothesis regarding AD pathophysiology, and they continue to provide evidence in its favor. 18

In cases of late-onset AD (LOAD ≥ 65 years of age), the importance of genetic factors has been proven by studies of monozygotic twins, which demonstrated a concordance of approximately 60 to 80%. 19 20 21

The genetics of AD can be understood as represented by rare mutations with autosomal dominant inheritance. Furthermore, there are polymorphisms or variations considered as risk factors. 19 20 21 22

In early-onset AD (EOAD) with onset of symptoms below age of 65 years, which represent about 10% of AD cases, an autosomal dominant inheritance pattern is observed in approximately 10%. These are caused by rare mutations with a penetrance of almost 100%. The main mutations are in the presenilin 1 and 2 genes, which are related to the abnormal cleavage of the amyloid precursor protein (APP) or in the APP gene itself. 22

On the other hand, LOAD follows a complex polygenic inheritance pattern, as occurs in arterial hypertension, diabetes mellitus, and coronary disease, in which variations in several genes cause an increased risk. 19 20 21

### *Apolipoprotein E* ( *APOE* )

The *apolipoprotein E* gene ( *APOE* ) presents normal polymorphisms with three alleles ( *ε* 2, *ε* 3, and *ε* 4) of variable frequencies in cognitively normal individuals in different populations, but the most frequent one is *ε* 3, with a frequency of approximately 79%, while *ε* 4 is 14% and *ε* 2 is 7% in Caucasian populations. The main genetic risk factor for LOAD, with a high odds ratio (OR), is the presence of the *ε* 4 allele of the *APOE* gene (APOE4). 23

The presence of the *ε* 4 allele increases the risk of AD with an OR near 3.68 (95%CI: 3.30–4.11) in Caucasian populations. Furthermore, ORs are higher in Asian populations and lower in Black and Latino populations. 19 21 23 When homozygous, the *ε* 4 allele greatly increases the risk of AD. 19 20 21 23 24

The presence of the *ε* 2 allele has a protective effect, with an OR close to 0.62 (95%CI: 0.46–0.85). In other words: it reduces the AD risk by almost 40%. 23

Homozygosity for the *ε* 2 allele is very rare in the Caucasian population, around 0.5%. In a neuropathological study with 5,000 subjects, the APOE2/2 genotype was associated with an extremely low likelihood of AD (OR: 0.13; 95%CI: 0.05–0.36) when compared to the most common APOE3/3 genotype. 23 In other words, persons with the APOE2/2 genotype had 87% lower likelihood of AD than those with APOE3/3. 19 20 21 23 24

It is not the objective of this article to review the complex underpinnings related to *APOE* and AD. It is known that this gene has many functions beyond lipid transport and that it interferes in many biological processes, including Aβ aggregation, tau pathology, neuroinflammation, cerebrovascular integrity, and others that may be relevant in the pathophysiology of this condition. 25

### Other genetic risk factors (common variants)

Many variants (or polymorphisms) with allele frequency >5% that are associated with AD have been described, but each one has a low OR, ranging between 1.05 and 1.25. Genome-wide association studies (GWAS) have, so far, identified 85 loci harboring common genetic variation associated with AD. Only a minority fraction of heritability—3.1% per the largest study to date—is explained by these common variants. 19 20 21 26

The simple determination of *APOE* alleles can currently calculate the risk with greater precision than these gene sets. 19 20 21 27

The genes in which variations have been found are related to many pathways compromised in AD, such as APP processing, neuroinflammatory responses, innate immunity, endocytosis, cytoskeletal transports, lipid metabolism, tau aggregation, and synaptic dysfunction. 19 20 21 27

Most studies have been carried out in Caucasian populations, so there is a need for studies in other populations to discover other susceptibility loci that may allow a better understanding of this disease's pathophysiology. 20 27

### Other genetic risk factors (rare variants)

Until recently, variations with very low allele frequency (<1%), called rare variants, had not been studied in detail. The discovery of a rare allele frequency variant in the triggering receptor expressed on myeloid cells 2 (TREM2) gene with OR ranging from 2.90 (95%CI: 2.16–3.91) to 5.05 (95%CI: 2.77–9.16) has motivated increasing interest in rare genetic variants in AD, having also highlighted the important role of microglia and neuroinflammation in this condition. 28 To verify them, it is necessary to perform GWAS in large populations and to apply recently available genome sequence reference panels. 26 27 28 29

Rare genetic variants have been described in 13 genes, some of which may increase while others may reduce the risk of AD. 26 29 They may have a relatively high effect when present.

An identified protective variant in the APP gene can decrease b-secretase cleavage by 40%, reducing the risk of AD by five times in individuals of Icelandic or Scandinavian descent. 30 31

A rare variant, known as APOE ?3 Christchurch, when in homozygosity, delayed the onset of clinical manifestations for more than 30 years and modified the pathological changes in a carrier of a pathogenic mutation in the presenilin-1 gene. 32

Genetic studies to verify the presence of rare variants have not yet been used in clinical practice, but the incorporation of these and other rare variants may allow a more accurate calculation of the PRS, and they should be evaluated in individuals of other ethnicities for adequate use of genetic information. 20

The relevance of studies in non-Caucasian populations is exemplified by the lower risk of AD in APOE4 individuals of African ancestry. This led to the identification of a single nucleotide polymorphism (rs10423769), 20 which was associated with an estimated 70% reduction in the risk of AD for APOE4 homozygotes. This discovery may be a therapeutic target for future investigations. 20

Returning to the issue of predicting AD (or PRS), it is necessary to consider that information obtained from one population, such as the Caucasian one, cannot simply be transferred to another. Many more studies are needed to reach a specific PRS for different populations or groups within the same population. This also applies to other diagnostic biomarkers, an aspect that should not be overlooked.

### Neuroinflammation

In recent years, there has been significant progress in understanding the pathophysiology of AD. From the phase now called biochemical, in which Aβ (β-amyloid peptide with 42 amino acids) plays the main role, comes the cellular phase in which microglia, seconded by astrocytes and oligodendrocytes, take the lead. 33

As mentioned, this knowledge was mainly due to the discovery that variants of the *TREM2* gene increase the risk of AD with an OR greater than 2.0. Other genetic studies with GWAS revealed that rare variants are located in susceptibility loci, of which a large group could be ascribed to pathways within the innate immune system regulation. 34 Several of the genetic loci associated with AD risk (or protective factors) contain genes with known roles in inflammation, the complement system and the immune response in general. 34 35

Microglia and their extensions around neuritic plaques have been identified many years ago in neuropathological studies. Briefly, one of the main current hypotheses is that microglia form a barrier that limits the spreading and neuronal toxicity of Aβ, delaying disease progression for many years through phagocytosis. 33 However, chronic activation of microglia causes its transition and the transition of astrocytes to proinflammatory phenotypes in which phagocytosis is impaired, causing accumulation of Aβ and of other proteins, cellular, and metabolic debris, as well as release of proinflammatory cytokines (interleukin-1 Beta [IL-1β], tumor necrosis factor-Alpha [TNF-α], and interleukin-6 [IL-6]) and chemokines, leading to neuroinflammation. 33 35 36 Chronic neuroinflammation has been revealed as a key aspect of CNS neurodegeneration in AD. 33 34 35 36

These studies reinforce the hypothesis that in autosomal dominant forms of AD and EOAD, there is an overproduction of Aβ, while in LOAD, the failure of Aβ clearance largely by microglia is a key factor in the disease pathogenesis. 27 Age-related failures in clearance may explain the associated presence of alpha-synuclein, TDP-43, and other proteins in LOAD, particularly in the older populations. 27

The upregulation of innate immune pathways is a process that may explain the effect of APOE4 as a risk factor for AD. Its expression in microglia and astrocytes is associated with the accumulation of cytoplasmic lipid droplets, defects in endolysosomal trafficking, and impaired mitochondrial metabolism, which causes the transition of microglia into a reactive state. 25

The presence of neuroinflammation in AD can be verified by biomarkers and PET tracers. The glial fibrillary acidic protein (GFAP) is elevated in response to astrocyte reactivity and soluble *TREM2* , which is elevated with microglial activation, making these the most studied fluid biomarkers of inflammation in AD. 37 38

Finally, PET tracers are used to target proteins which are known to be expressed in activated microglia and astrocytes, such as translocator protein 18 kDa (TSPO), and monoamino-oxydase B (MAO-B) tracer, though they lack specificity for the diagnosis of AD. There is ongoing research to find new PET tracers of inflammation. 39

### Diagnostic accuracy

The diagnosis based on that heavy amount of information about clinical data, neuroimaging, plasma (and other fluid) biomarkers, and genetics will most likely identify different forms of AD, which may have different evolutions and treatments.

Therefore, the diagnosis will be individualized and could make use of AI, with methods such as machine learning (ML) being able to build optimal predictive models to distinguish these different forms of AD. 40 However, ML may cause bias when applied to individuals of different populations from those where the algorithmics were devised. 41

### Timing of the diagnosis

The complex question we ask ourselves today is whether there is value to diagnosing AD when there is no treatment with clinical efficacy. 1 2 If evaluated from the point of view of scientific logic and medical procedures, everyone would agree that diagnosis should always precede treatment. The real question is when to transfer diagnostic methods from research into clinical practice when there are no adequate or uncontroversial treatments.

The diagnosis of asymptomatic AD based exclusively in plasma biomarkers is possible according to new criteria, 3 which also included in Box 4 that “the clinical use of AD biomarkers is presently intended for the evaluation of symptomatic individuals, not cognitively unimpaired individuals.” 3

For the International Working Group (IWG) asymptomatic individuals with positive plasma biomarkers should be diagnosed either as “at risk for AD dementia” or as “presymptomatic AD dementia” according to the biomarker profile, which may suggest that the underpinning pathological processes are in the initial phase (at risk for AD dementia) or they are in such an advanced stage that clinical symptoms will probably manifest in the near future (presymptomatic AD dementia). 4

Both positions are correct and are not irreconcilable. The IWG proposal is much more appropriate for communicating diagnoses in clinical practice. Furthermore, AD's pathological changes have been identified by many studies in individuals who died with normal cognition. 42

This is the moment we are living in today, and it will be overcome by the rapid advancement of new therapeutic modalities, as presented below.

## TREATMENT

### Prevention of dementia

Systematic reviews and meta-analyses have shown that by reducing risk factors for vascular dementia and increasing cognitive and physical reserve across one's lifespan it is possible to reduce its incidence. In the last revision of the Lancet Comission on dementia, 14 risk factors were identified. Tracking these potential variables would reduce the incidence of dementia by 45%. 43

In a previous article of this series, 44 comments were made on the FINGER study, a multidomain intervention for dementia prevention. 45 Although this study and other similar ones, such as the U.S. POINTER study, 46 have the objective of preventing dementia, not just AD, and are primarily related to the prevention of vascular diseases, one should take into account that this multidomain intervention may reduce the risk of AD by also interfering on epigenetic mechanisms, genomics, proteomics, and metabolomics. 27 These interventions, proposed as secondary prevention of AD dementia (and for dementia of other causes), are very important and will continue and even improve with the additions of future interventions to correct other risk factors that may be discovered, as well as to improve lifestyles. Along with interventions in lifestyle, cognitive training is also included. In the LatAm-FINGER study, 47 cognitive exercises are implemented using BrainHQ (Posit Science Corp.), a computerized program for cognitive training. 48

Recent reviews have suggested that the use of unsupervised computerized cognitive stimulation programs may lead to cognitive gains, especially among cognitively unimpaired or mildly impaired participants. 49 50 Learning and practicing with mnemonic strategies should happen on a large scale through online platforms. Also, significant evidence suggests that cognitive stimulation therapy, based on stimulating and meaningful activities carried out in small groups, may lead to stabilization in cognitive decline, as well as improvement in mood and in activities of daily living. 51 52 53

Noninvasive brain stimulation (NBS) is another intervention for secondary prevention of cognitive decline or dementia, which has been extensively studied in recent years and for which there is an expectation of success. Excellent reviews of NBS and secondary prevention of cognitive decline and dementia for at risk individuals were recently published. 52

### Drug treatments

In 2021, aducanumab, a monoclonal antibody directed to amyloid-beta protein (mAβ), was approved. 54 This involved nearly 20 years of research, with many unsuccessful clinical trials (CTs). More recently, 2 mAβs were approved: lecanemab and donanemab. 55 56

These approvals were very important for two reasons. The first and most important one was the partial confirmation of the amyloid cascade hypothesis. 18 27 In other words, the reduction of Aβ protein deposits in the brain parenchyma demonstrated a statistically significant reduction in the rate of disease progression compared to placebo. 27 55 56

The second is that this discovery has brought renewed enthusiasm for research into new drugs. The recent increase in phase 1 trials, which nearly doubled compared to 2024, reflects the increased investment in research into treatments for AD. 57 58 The predicted increase in research funding, both from the pharmaceutical industry and other private and public institutions, will certainly contribute to the development and approval of new effective drugs. This is the most important aspect of the recent approvals of mAβ, which will certainly make a significant difference in near and ultimate future of AD treatment. 58

It is important to recognize, however, that the results were statistically significant, but the effect size was very small. 59 60 61 The minimum clinically-important difference (MCID) was not reached, 1 61 and side effects were clinically significant and potentially serious. 1 55 56 The enormous importance of discovering these drugs for research and advancement of treatments for AD are not reflected in the importance of these drugs for current clinical use.

However, we do not know whether the effect size of these mAβ will reach the MCID when used in very early pathological phase of AD. There is an ongoing trial using donanemab in individuals with preclinical AD with diagnosis established through plasma biomarkers, which may be concluded in 2027 (TRAILBLAZER-ALZ 3). 62

The treatment of any disease, especially complex ones like AD, depends on knowledge of its pathophysiology. The treatment of AD will advance along with greater understanding.

### Pathways for treatment

At the risk of oversimplifying such a complex problem, we will comment on five pathways that perhaps have not been sufficiently explored, for different reasons. We also are going to highlight a few CTs that are trying to delve into these pathways.

#### *Aging*

The first of these is that the main factor responsible for AD is aging. And for reasons that are only briefly discussed here, AD has often been placed at the margins of aging, with the idea that it would be possible to understand and treat AD without taking aging into account. 63

There is now a renewed understanding of the pathophysiology and treatments that are also part of geroscience, an interdisciplinary field of study that investigates the relationship between aging and diseases occurring during this period of life. 63 64 The very optimistic geroscience hypothesis can be resumed in this sentence: “… targeting aging we can prevent or delay multiple chronic diseases and death simultaneously.” 65

Therapies that inhibit aging biology, such as caloric restriction, metformin, and drugs that selectively clear senescent cells (or senolytics), may slow the development and progression of AD, as well as functional decline in humans. For example, chronic, sterile inflammation occurs in many diseases in the aging process and is caused by a variety of inflammatory stimuli that include senescent cells, endogenous cell debris, and misfolded proteins. 66 Drugs and interventions that have been used in studies in the field of geroscience as anti-inflammatory agents, bioenergetic and metabolic therapies are among the well represented classes in the AD pipeline, being applicable to age-related conditions including the one studied here. 57

Recently, a phase 3 CT with semaglutide, 57 58 a glucagon-like peptide-1 (GLP-1) receptor agonist, failed to demonstrate a statistically significant reduction in progression in early AD patients, according to a press relase. 67

Metformin, a drug used for treating patients with type 2 diabetes, is the most interesting one among geroscience studies. Clinical observations have been influential in generating support for the hypothesis of anti-aging effect of metfomin. These include pronounced anticancer and cardioprotective benefits compared to other antidiabetic treatments, and an observation of metformin use in type II diabetes being associated with better survival than that of the general population. 68 69

A recent study in cynomolgus macaques showed that metformin diminished the multidimensional biological age and alleviated neuronal aging in male primates. 70

The targeting aging with metformin (TAME) is investigating the anti-aging effect of metformin in a large human cohort of 3 thousand individuals. 70 The MET-FINGER, a study combining multimodal lifestyle interventions and metformin was launched in 2024, 71 showing that there is considerable confidence on the anti-aging effect of metformin.

#### *Apolipoprotein E (APOE)*

The second is the APOE pathway. It has been known for over 30 years that its polymorphism is the main genetic factor for LOAD, but progress in this field has been very small regarding the diagnosis and treatment of AD. 72

Recently, interest in *APOE* has increased. We will highlight the LX1001, a recently closed CT, which did not present final data yet. 62 In short, it consisted on introducing an adeno-associated virus (AAV) gene transfer vector expressing the complementary deoxyribonucleic acid (cDNA) coding for human APOE2, by lumbar puncture, into patients with the APOE4/4 genotype with AD in the MCI phase, as well as mild or moderate dementia. 73 The insertion of the Christchurch mutation protects against APOE4-driven tau pathology, neurodegeneration, and neuroinflammation in transgenic mice. 74 As far as we know, the insertion of the polymorphism rs10423769 in the *APOE* *ε* 4 has not been evaluated in research with transgenic animals yet.

#### *Neurofibrillary tangles*

It is well known that the correlation between the presence of neurofibrillary tangles, whose main component is hyperphosphorylated tau protein, and clinical symptoms is greater than that between the presence of Aβ deposits in neuritic plaques. 10 11 Tau protein is intracellular, which makes it difficult for drugs that could inhibit hyperphosphorylation and acetylation to act on it, as these are posttranslational phenomena. Other strategies may begin to overcome this obstacle.

A recent CT used an antibody (E2814 or etalanetug) against the microtubule-binding region (anti-MTBR) of tau protein. 75 This region is considered important for seeding of tau protein between neurons, which is responsible for spreading through cognitive networks. 75 This study is already in phase 3 and the Dominantly Inherited Alzheimer's Disease—Treatment Unit (DIAN-TU) is assessing the combination of lecanemab with this anti-MTBR antibody. 57 58

#### *Genetics*

The fourth pathway is related to the relatively low use of genetic techniques in diseases in which genetic alterations are so important. Besides the already mentioned CT LX1001, there is the BIIB080 in phase 1/2 that consists on using antisense oligonucleotides (ASOs) introduced into the cerebrospinal fluid (CSF) by lumbar puncture with the aim of inhibiting the translation of tau protein by the MAPT gene. 76 Recently, the U.S. Food and Drug Administration (FDA) has granted Fast Track designation to this drug. 77 Therefore, there are 2 CTs that are highly likely to overcome these last two obstacles.

Gene editing methods are being used only in research and are not approved for use in humans for ethical reasons and due to risks they may present. We are still far from gene-edited treatment for cases with mutations in PS1, PS2, and APP, but gene editing for the E4/E4 allele of *APOE* to E3/E2 is already in research in animals. 78 As previously mentioned, there are several recently described rare variants that reduce the risk of AD. It is highly probable that they will be used as prevention in the near future. For instance, insertion of coding mutation A673T (the Icelandic mutation) in the APP gene of transgenic mice downregulated β-cleavage, reducing amyloid pathology in the brains of these animals, having also decreased neuroinflammation and neuritic alterations. 79 The possibility of using this method for patients with known dominant inherited AD is probably not far in the horizon.

#### *Animal models*

Finally, the lack of animal models is a major limiting factor; AD was considered an exclusively human disease until neuropathological changes with neuritic plaques were discovered in chimpanzees; 80 and more recently in capuchin 81 and rhesus monkeys. 82 Nonhuman primates (NHPs) share many similarities with humans, and aged NHPs with memory impairments can be used for experimental therapies. Also, transgenic NHPs may be better than rodents to study pathophysiology and treatment of AD. Human stem cell derived brain organoids are a different model that may advance understanding and drug development in AD. 83

### Clinical trials

To understand the current stage of research, which will certainly have an impact on nearest future, we use the most recent review conducted by Cummings et al., 57 who each year present the pipeline of drugs registered in a clinical research registry (clinicaltrials.gov) maintained by the US National Library of Medicine of the National Institutes of Health, which includes almost all ongoing CTs for AD.

In that study, CTs are divided according to one of the three phases, as well into three groups according to the treatment targets, which are: disease-targeted therapies (DTTs), cognitive enhancers, and treatments of neuropsychiatric symptoms. 57

Cognitive enhancers and treatments of neuropsychiatric symptoms are very important, especially for the quality of life of patients who have already reached the dementia stage, but they will not be discussed in this review.

Focusing on DTTs, the group is subdivided into biological therapies (such as monoclonal antibodies, antisense oligonucleotides, gene therapy etc.) and small molecules. At that latest review, there were 138 drugs in CTs, 102 (73.9%) of which were DTTs. Among them, 60 were small molecules and 40 biological DTTs. 57

For the nearest future, we should concentrate on DTTs in phase 3, which is the last phase of research before submission to regulatory agencies. There are 20 DTTs in phase 3, 7 targeting Aβ-related pathophysiology, 3 related to neuroinflammation/immune processes, two addressing metabolism and bioenergetics, 2 targeting proteostasis/proteinopathy, and 1 agent each targeting tau-related processes, growth factors and hormones, neurogenesis, neurotransmitter receptor, vasculature, and one with combination therapy targeting Aβ- related pathophysiology with tau-related processes. 57

Furthermore, four of the treatments targeting Aβ-related pathophysiology in phase 3 are for individuals in preclinical AD, a condition where mAβ may have more effect than in MCI or mild dementia due to AD. 57

For a relatively later future it is important to know that in phases 1 and 2, there are 23 CTs addressing Aβ-related pathophysiology, 22 related to neuroinflammation/immune processes, 20 to neurotransmitter receptors, 14 to tau-related processes, 8 to synaptic plasticity and neuroprotection, and 7 to metabolism and bioenergetics. Compared to the drugs in phase 3, there has been a a recent increase in research on inflammatory and tau-related processes. 57 58

There is one CT with a small molecule (VG-3927), in phase 1, which is a TREM2 agonist. 84 Sodium oligomannate, 85 a drug with possible anti-inflammatory effects which works through the gut microbiome, was approved and is in use in China. 85

A CT addressing Aβ-related pathophysiology, still in phase 2, 51 investigates the effects of trontinemab. 86 This new drug was projected to solve the difficulty for mAβ to cross the BBB. Trontinemab is a conjugate of gantenerumab (mAβ) with a second antibody targeting the transferrin receptor (TfR), which has the ability to bind to the transferrin molecule, a transmembrane protein that allows the passage of macromolecules across the BBB via receptor-mediated transcytosis. In NHP, trontinemab showed approximately 4 to 18-fold increased exposure in multiple brain regions when compared with gantenerumab alone. 86

### Main challenges for CTs

The biggest challenges in performing CT scans for AD are time and cost. Currently, DTTs take more than 9 years from preclinical development to regulatory approval, and the cost exceeds $5 billion. 57 58

The need of neuropsychological and/or functional tests to verify the effectiveness of an AD drug is one of the reasons for long delay of the CTs. Remote measurement technologies for assessing functional status in AD may be auxiliary methods for evaluation of disease progression. 87 Of course, neuropsychological and functional indexes are the main targets of these trials, but surrogate markers of pathological evolution are also needed. 57 58

In slow progression diseases, it is very difficult to demonstrate the effect of a drug, particularly now, when CTs are being proposed for the preclinical stages of AD. In the 2025 AD pipeline, 27% of the CTs have a biomarker as primary outcome, which is good news. 57 Probably, the evolution of the Braak stages evaluated either by tau-PET or plasma biomarkers will be used in nearest future. Furthermore, light chain neurofilament, GFAP, biomarkers of synaptic degeneration are other possible disease monitoring biomarkers.

### Individualization of treatment

According to the concept of precision medicine, AD treatment will be personalized. 21

Treatments in the future will be administered before or just after the detection of the first pathological biomarkers. Individuals with a higher genetic risk identified will likely be monitored with biomarkers from adulthood or even earlier. Genetic treatments, like insertions of protective genetic variants, will also be used.

The hypothesis that a ‘silver bullet’ can treat a complex disease like AD has already been abandoned. 88 In addition to individual characteristics, the stage at which the disease is found will also require very different treatments. Treatments that reduce the concentration of Aβ in its various forms (oligomers or protofibrils) will be more efficient in the very early stages of the disease, and anti-tau drugs will be efficient from the early stages and throughout the course of evolution to prevent the spread of tau protein. Anti-inflammatories will be very important, but with different drugs for each AD pathological stage.

Treatments that can slow or stop disease progression once it has begun will also be very important. Living with stable MCI or stable mild dementia in AD is compatible with a reasonably good quality of life. With advances in IT, new applications that may be much more useful than current ones can make life easier. Current applications inform us about semantic data (semantic memory) but not about autobiographical or episodic memory, which are the most impaired in AD, from its earliest symptoms. Combining reduced or halted AD progression with autobiographical (and semantic) memory devices will improve the quality of life even more for those already in the early stages of AD.

Challenges for low- and middle-income countries (LMICs), as presented in the **Supplementary Material** ( https://www.arquivosdeneuropsiquiatria.org/wp-content/uploads/2026/02/ANP-2025.0375-Supplementary-Material.docx ).

In conclusion, in nearest future, the diagnosis and treatment of AD will be very different. Genetic data will be incorporated into the current methods. Diagnosis will be individualized, since the causes of AD will be slightly or even very different for each patient or group of patients. Furthermore, it will be made in the stages prior to clinical manifestations. The diagnosis of risk factors for AD, based on the preponderant role of genetics, will be made very early. Also, the complexity caused by the large number of variables will be facilitated with the use of AI.

The prevention of AD and other degenerative diseases that accompany it during the aging process will be possible with better control of environmental and lifestyle risk factors, drugs, cognitive training, and genetic therapy. The relationships between AD and aging will be better understood, opening possibilities for intervention in aging itself.

As the pathophysiology of AD is diverse, treatment will be individualized according to the stage of the disease, with the use of multiple drugs with multiple targets. The treatment of choice will also depend on each doctor's experience, supported, but not directed, by AI. We will begin to make greater use of surrogate markers to monitor results. Treatments to decrease the velocity or arrest the evolution of MCI or mild dementia caused by AD will become available. The possibility of reversing dementia once it has set in probably will have to wait for a more distant future.

From there, we can give free rein to our imagination about what an elderly human being of the future would be like, with the clarity of mind provided by a brain free of AD, combined with experience (and, if it is possible, also free of the ‘slings and arrows of outrageous fortune’; Shakespeare W. Hamlet, Act III, Scene I). Human history will reach a turning point.
